# The contribution of helicopter emergency medical services in the pre-hospital care of penetrating torso injuries in a semi-rural setting

**DOI:** 10.1186/s13049-021-00929-8

**Published:** 2021-08-04

**Authors:** M. Gavrilovski, J. E. Griggs, E. ter Avest, R. M. Lyon, on behalf of Air Ambulance Kent Surrey Sussex

**Affiliations:** 1 Air Ambulance Kent Surrey Sussex Trust, Rochester City Airport, Maidstone Road, Kent, ME5 9SD UK; 2 Department of Health Sciences, University of Surrey, Guildford, GU2 7XH UK; 3 University Medical Center Groningen, Department of Emergency Medicine, University of Groningen, Groningen, The Netherlands

**Keywords:** Penetrating trauma, County lines, HEMS, Pre-hospital

## Abstract

**Background:**

Although the merit of pre-hospital critical care teams such as Helicopter Emergency Medical Services (HEMS) has been universally recognized for patients with penetrating torso injuries who present with unstable physiology, the potential merit in patients initially presenting with stable physiology is largely undetermined. The ability to predict the required pre-hospital interventions patients may have important implications for HEMS tasking, especially when transport times to definitive care are prolonged.

**Methods:**

We performed a retrospective cohort study of patients who sustained a penetrating torso injury and were attended by the Air Ambulance Kent Surrey Sussex (AAKSS) over a 6-year period. Primary outcome was defined as the percentage of patients with penetrating torso injuries requiring HEMS-specific interventions anytime between HEMS arrival and arrival at hospital. Secondary outcomes were the association of individual patient- and injury characteristics with the requirement for HEMS interventions.

**Results:**

During the study period 363 patients met inclusion criteria. 90% of patients were male with a median age of 30 years. 99% of penetrating trauma incident occurred more than 10-min drive from a Major Trauma Centre (MTC). Presenting GCS was > 13 in 83% of patients. Significant hemodynamic- or ventilatory compromise was present in more than 25% of the patients. Traumatic cardiac arrest was present in 34 patients (9.4%), profound hypotension with SBP < 80 mmHg in 30 (8.3%) and oxygen saturations < 92% in 30 (8.3%). A total of 121 HEMS-specific interventions were performed. Although HEMS-specific interventions were associated with presenting physiology (TCA OR 1.75 [1.41–2.16], SBP < 80 mmHg (OR 1.40 [1.18–1.67] and SpO_2_ < 92% (OR 1.39 [1.17–1.65], a minority of the patients presented initially with stable physiology but deteriorated on route to hospital and required HEMS interventions (*n* = 9, 3.3%).

**Conclusion:**

HEMS teams provide potentially important contribution to the pre-hospital treatment of patients with penetrating torso injuries in rural and semi-rural areas, especially when they present with unstable physiology. A certain degree of over-triage is inevitable in these patients, as it is hard to predict which patients will deteriorate on route to hospital and will need HEMS interventions. The results of this study showing a potentially predictable geographical dispersion of penetrating trauma could inform multi-agency knife crime prevention strategy.

## Background

Trauma can be a time sensitive condition [1]. This is reflected by the golden hour concept dating from 1975, which is still ingrained in trauma systems and national field triage guidelines used by emergency medical services [2]. As a result, Emergency Medical Services (EMS) often try to minimize scene time for trauma patients [3, 4], even though most studies have not demonstrated a clear association between pre-hospital time and patient outcome [2, 5–7]. For some subgroups of trauma patients however, prolonged scene times have been associated with an increased mortality [8].

One of these subgroups comprises of patients with penetrating trauma to the torso. These patients pose a significant challenge to pre-hospital care providers, as bleeding from underlying vascular structures is non-compressible [9]. In the main, these patients benefit from expeditious transport to a specialist trauma centre [3, 10]. This is especially true when advanced critical care teams with the capability to perform pre-hospital interventions such as blood product transfusion, thoracostomies or thoracotomy are not immediately available [4, 11].

However, patients with penetrating trauma of the torso are a highly heterogenous group of patients [12]. Some patients will have critical injuries resulting in rapid clinical deterioration (tension pneumothorax, cardiac tamponade or a large artery laceration). These patients are likely to deteriorate before the arrival of critical care teams. Most will benefit from critical interventions on scene, and all of them will benefit from expedited transport to definitive care [10]. Other patients, however, do not demonstrate such a rapid deterioration as a result of their injuries, but given the extent and location of their injuries still have the potential to do so [13].

Although the merit of critical care teams such as Helicopter Emergency Medical Services (HEMS) has been recognized for patients with penetrating torso injuries who present with unstable physiology, the potential merit in patients initially presenting with stable physiology is largely undetermined. Being able to predict the clinical course of these patients may have important implications for the tasking of HEMS teams, especially when transport times to definitive care are prolonged [8].

Previous research has demonstrated that clinical examination alone is a poor predictor of both severity and depth of penetrating injuries [14, 15]. Therefore, in this study we aim to describe the patient demographics and clinical interventions required in patients with penetrating torso injuries treated by a semi-rural HEMS service.

## Methods

### Study design and setting

A retrospective cohort study was performed of all patients with penetrating torso injuries attended by Air Ambulance Kent Surrey Sussex (AAKSS) during a 6-year period (1 January 2014 to 31 December 2019). AAKSS is a HEMS service covering three counties in the southeast of England with a resident population of 4.5 million and transient population of up to 8 million. Doctor-paramedic teams respond in either a helicopter or response car from one base. The service attends approximately 2000 patients per year. Most patients attended by the HEMS service are first seen by a ground ambulance crew and/or a critical care paramedic.

A Critical Care Paramedic and HEMS dispatcher screen and task the critical care resource simultaneously with a ground ambulance crew/critical care paramedic from the Emergency Operations Centre. The tasking algorithm was devised internally, and is previously published [16]. Activations are categorised as: immediate (Grade 1), interrogated (Grade 2) or crew request (Grade 3 and 4). Immediate dispatch is triggered by pre-determined criteria. Interrogated dispatch is triggered where subsequent clinical information is reviewed, and HEMS dispatch agreed. Both immediate and interrogate dispatches are based on mechanism of injury, clinical condition of the patient and geographical location. A crew request can be activated by crews on scene.

Penetrating trauma attracts a grade 1 dispatch when there is indication of a persistent decreased level of consciousness and/or collapse, or high impact penetrating trauma to the chest with shortness of breath, difficulty in breathing and/or collapse.

### Treatment of penetrating injuries and patient disposition

In-line with AAKSS Standard Operating Procedure on penetrating torso trauma, time to definitive care is paramount. When physiology allows, the HEMS team facilitates expedited transfer to definitive care either by ground escort or air transfer, without further interventions on scene.

When patients present with shock physiology, both obstructive shock causes are excluded and treated, as is hemorrhagic shock. FiO_2_ is maximized with a non-rebreather mask, and, if co-operative, the patient will self-ventilate. Tranexamic acid is administered, and where verbal contact is lost, or when the systolic blood pressure (SBP) is < 80 mmHg (when verbal contact is not possible, e.g. in intubated patients), volume therapy with warmed packed red blood cells (PRBC) and freeze-dried plasma (FDP) is initiated. Up to four units of PRCs are stored in a CRĒDO CUBE™ (Series 4, 2 l Insulation 15, VIP Golden Hour) and four units of plasma (Lyoplas) are transfused using a Qinflow™ fluid warmer. Transfusion is titrated to systolic blood pressure (SBP) and/or Glasgow Coma Score [GCS], aiming for cerebration and/or SBP > 80 mmHg. When there is penetrating trauma to chest, abdomen or limbs with loss of cardiac output on scene or on route to hospital, and where tension pneumothorax is excluded, resuscitative thoracotomy is performed if cardiac arrest occurred withing the preceding 10 min.

All patients with head, neck, chest, axillae and groin wounds are triaged to an MTC irrespective of clinical state. Patients with limb wounds are triaged to a Trauma Unit (TU), unless significant neurovascular compromise is anticipated. A pre-alert to the receiving hospital is made at the discretion of the pre-hospital team in order to trigger a predefined in-hospital major hemorrhage protocol based on presenting pre-hospital clinical signs & physiology, ensuring blood and clotting factors are immediately available upon arrival [17, 18].

### Study population

Patients were eligible for participation in the study if they had sustained a penetrating injury to the torso (chest, abdomen or junctional zones [neck, axilla and groin]) for which they were attended by AAKSS HEMS during the study period. Location of penetrating injury was determined based on the notes entered by the treating physician in mandatory entry fields of the bespoke electronic patient clinical record system (HEMSbase 2.0, Medic One Systems Ltd., UK).

### Data acquisition

The following variables were collected from HEMSbase: demographic descriptors (age [years], gender [male/female]), geographical descriptors [postcode], injury descriptors [injury mechanism, anatomical site of injury, mission timings (time of injury (day [0700–1900] or night [1900–0700], exact 999/112 time, arrival on scene time, scene departure time, arrival at hospital time), patient physiology upon presentation of HEMS (presence of profound hypotension defined as a SBP < 80 mmHg) and tachycardia (defined as a HR > 100 bpm), presenting GCS, presenting prehospital Lactate (mmol/l) (if measured), HEMS interventions (finger thoracostomy, intercostal chest drain insertion, blood product administration (PRBC/FDP), pre-hospital emergency anaesthesia [PHEA]), intubation without drugs by HEMS, resuscitative thoracotomy [RT]), and patient result (carry in the aircraft [Air], ground escort [GE] with HEMS team, ground assist [GA] with local ambulance crew, discharged from scene or pronounced life extinct (PLE). Thoracostomies are counted per thoracostomy and not reported where a thoracotomy was subsequently performed. To get a better overview of the geographical distribution of penetrating injuries, heat-mapping charts of incidents recorded were generated using Maptive [19].

### Clinical endpoints

The cohort of patients will be subdivided into those requiring HEMS specific interventions and those who didn’t. The need for HEMS specific interventions was deemed a worthy surrogate for injury severity, thereby clinical course in our study population.

#### Primary endpoint

The percentage of patients with penetrating torso injuries attended requiring specific HEMS interventions anytime between HEMS arrival and presentation in hospital.

#### Secondary endpoint

The association of individual patient- and injury characteristics with the requirement for HEMS interventions.

### Ethical considerations

This project met National Institute for Healthcare Research (NIHR, UK) criteria for service evaluation and formal ethical approval was therefore not required. The project was approved by the AAKSS Research & Development Committee.

### Statistical analysis

Descriptive statistics are given as mean [95% CI] or median [IQR]. Comparisons across groups were made using Fisher’s exact test, Mann-Whitney U test and the Student’s t-test where appropriate. When three or more groups were present, nominal data were compared using Kruskal-Wallis test. Univariate correlation analysis with calculation of Spearman correlation coefficients was performed to evaluate the association of injury- and patient characteristics with the occurrence of physiology demanding HEMS interventions. Subsequent multivariable logistic regression analyses with calculation of odds ratio’s (OR) determined which factors were independently related to the primary outcome measure. A *p*-value < 0.05 was regarded as statistically significant. All statistical analyses were conducted using SPSS 26.0 for Mac.

## Results

### Study population

**Fig. 1:**
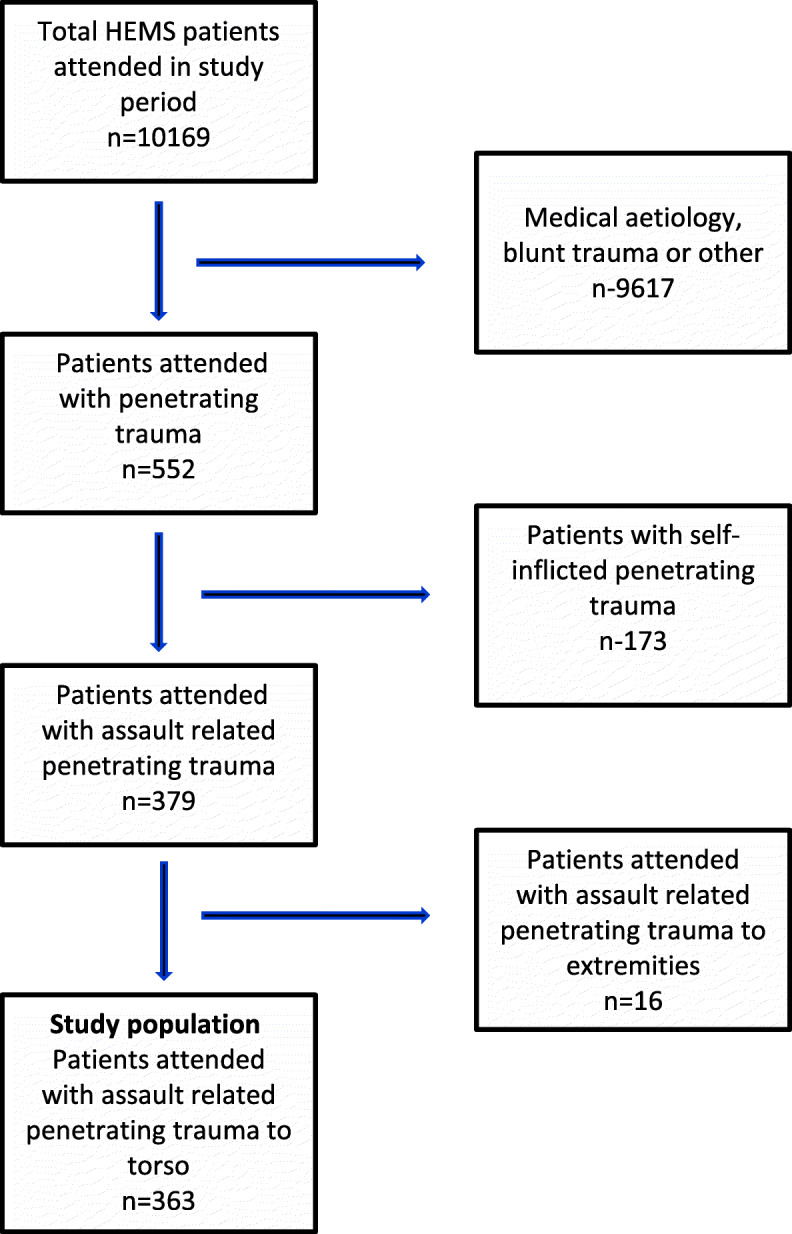
Flow chart of study population

During the study period AAKSS was tasked to 10,169 patients. Nine thousand six hundred seventeen patients (96%) sustained an injury by blunt trauma or were attended for medical (non-trauma) reasons. Five hundred fifty-two patients were attended as a result of sharp object penetrating trauma, of which 173 were self-inflicted. Sixteen patients out of the remaining 379 had isolated limb injuries, leaving 363 patients with sharp object penetrating torso injury as a result of an assault. Patient inclusion is shown below in Fig. 1.

### HEMS tasking and timings

**Fig. 2:**
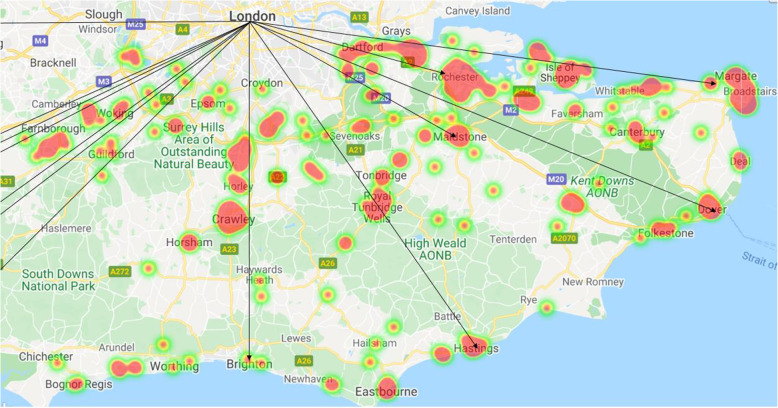
Heat map showing geographical distribution of penetrating trauma over a 6-year period in relation to drug-trafficking county lines, for the region served by AAKSS (southeast England). Red shading depicts areas of increased incidence of penetrating injuries attended by AAKSS across the geographical region. Black arrows represent the county lines drug supply routes across southeast England, stemming from the English Channel towards London. County lines are adapted from Coomber and Moyle (2018)

Geographical distribution of penetrating trauma over the 6-year study period exhibited a specific pattern which mirrored existing drug trafficking county lines (Fig. 2). The vast majority (99%) of penetrating trauma occurred more than 10 min drive from an MTC. Overall, the median [IQR] time to scene from 999/112 call was 39 [29–50] minutes and the median time to hospital was 1 h 30 [88–97] minutes (Table 1). The HEMS team was quicker on scene during daytime compared to nighttime (37 [19–31] mins vs 41 [20–35], *p* = .017), but the overall time to definitive care was not shorter (90 [62–106] mins vs 87 [47–93] mins, *p* = .958).

**Table 1:** Patient and injury descriptors of patients with penetrating torso injuries attended by AAKSS during a 6-year period stratified by the need for HEMS specific interventions

	ALL	HEMS interventions	No HEMS interventions	*P* value
TOTAL [n,%]	363	54 [14.9]	309 [85.1]	
Demographics				
Male [n, %]	326 [89.8]]	43 [13.2]	283 [86.8]	.349
Age [median, IQR]	30 [23–42]	30 [21–44]	31 [23–42]	.531
< =16 [n,%]	11 [3.0]	2 [18.2]	9 [81.8]	.272
17–25 [n,%]	114 [31.4]	15 [13.2]	99 [86.8]	.336
26–45 [n,%]	164 [45.2]	19 [11.6]	145 [88.4]	.100
46–65 [n,%]	60 [16.5]	13 [16.7]	47 [78.3]	1.00
> 65 [n,%]	14 [3.9]	5 [35.7]	9 [64.3]	.132
Job descriptors				
Daytime [0700–1900] [n,%]	155	29 [18.7]	126 [81.3]	.009
Night-time [1900–0700] [n,%]	208	25 [12.0]	183 [88.0]	.009
999-scene [hours:mins]	00:39	00:37	00:39	.583
999-hospital (hours:mins)	01:30	01:47:00	01:29:00	.283
Presenting physiology				
GCS [median]	15	4	15	
14–15	301 [82.9]	20 [6.6]	281 [93.4]	<.001
9–13	12 [3.3]	5 [41.7]	7 [58.3]	<.001
3–8	35 [9.6]	28 [80.0]	7 [20.0]	<.001
TCA [n,%]	34 [9.4]	27 [79.4]	7 [20.6]	<.001
Hypotension (SBP < 80 mmHg) [n,%]	30 [8.3]	16 [53.3]	14 [46.7]	<.001
Tachycardia (HR > 100 BPM) [n,%]	111 [30.6]	17 [15.3]	94 [84.7]	<.001
SpO2 < 92% [n,%]	30 [8.3]	12 [40.0]	18 [60.0]	<.001
RR > 30 [n,%]	32 [8.8]	10 [31.25]	22 [68.75]	<.001
Lactate (mmol/l [n,%])	26 [7.2]	4 [15.4]	22 [84.6]	.907

*GCS* Glasgow Coma Score; *TCA* Traumatic Cardiac Arrest; *SpO*_*2*_ Oxygen saturation; *RR* respiration rate

### Patient characteristics

Approximately half of the patients were seen during daytime hours (*n* = 155, 42.7%) and the other half during night-time operations (*n* = 208, 57.3%). Most patients were male (89.8%, *p* < .05) and the majority of the patients were relatively young (median age of 30 years [IQR 23–42]). Presenting GCS was > 13 in 301 (82.9%) of patients. A minority but significant number of patients (*n* = 34, 9.4%) went into TCA either before or after arrival of the HEMS team and 30 patients (8.3%) exhibited profound hypotension (SBP < 80 mmHg) before arrival in hospital. Respiratory distress was present in a minority of patients, with 30 patients presenting with SpO_2_ < 92% (8.3%) and 32 patients with a RR > 30 (8.8%). Lactate levels were measured in 26 patients and were elevated (> 2.0 mmol/l) in the majority (85%) (Table 1).

### Patient conveyance and disposition

HEMS conveyed the majority of patients (78.5%) to hospital. Most patients (*n* = 210, 57.9%) were conveyed by ground escort, with 75 patients (20.7%) being transferred by air. Most patients were conveyed to an MTC, with only a small proportion being triaged to TU or to a Local Emergency Hospital (LEH). Thirty-one patients were pronounced life extinct (PLE) at the scene (Table 2).

**Table 2:** HEMS conveyance and patient disposition of patients with penetrating torso injuries

	ALL	HEMS interventions	No HEMS interventions	*P* value
Patient conveyance	**363**	**54**	**309**	
Air	75 [20.7]	12 [16.0]	63 [80.4]	.470
Ground escort	210 [57.9]	18 [8.6]	192 [91.4]	.002
Ground assist	47 [12.9]	0 [0]	47 [100.0]	<.001
PLE	31 [8.5]	24 [77.4]	7 [22.6]	<.000
Patient disposition				
MTC	293 [80.7]	29 [9.9]	264 [90.1]	.001
TU	31 [8.6]	1 [3.2]	30 [96.8]	.006
LEH	3 [0.8]	0 [0]	3 [100.0]	.557
PLE	31 [8.6]	24 [77.4]	7 [22.6]	.000
Self-discharged	5 [1.4]	0 [0]	5 [100.0]	.001

*PLE* Pronounced Life Extinct; *MTC* Major Trauma Centre; *TU* Trauma Unit; *LEH* Local Emergency Hospital

### HEMS interventions

HEMS interventions were performed relatively infrequently. 54 of 363 (14.9%) patients received a total of 121 HEMS interventions. Thoracostomies were performed most often (*n* = 31) with other interventions such as intercostal chest drain insertion, pre-hospital emergency anesthesia (PHEA), blood product administration, thoracotomy, and intubation without drugs being performed less frequently (Table 3). The number of patients in whom HEMS interventions were performed was consistent over the 6-year study period (*p* = .132).

**Table 3:** HEMS specific interventions performed in patients with penetrating torso injuries

HEMS Interventions	*n* = 121
Thoracostomy n[%]	31 [25.6]
Intercostal chest drain n[%]	9 [7.4]
PHEA n[%]	10 [8.3]
Blood products n[%]	34 [28.1]
*PRBC (units)*	*76*
*FDP (units)*	*47*
Thoracotomy n[%]	18 [14.9]
Intubation, without drugs n[%]	19 [15.7]

*PHEA* pre-hospital emergency anaesthesia; *PRBC* packed red blood cells; *FDP* freeze-dried plasma

### Association of patient- and injury characteristics with the requirement for HEMS interventions

Patients receiving HEMS interventions required these based on presenting physiology. They had either a compromised circulation (TCA or SBP < 80 mmHg, *n* = 43), or isolated compromised ventilation (RR > 30 or SpO2 < 92% with suspicion of significant pneumothorax, *n* = 2). 27 out of 34 patients in TCA and 16 out of the 30 patients presented with hypotension (SBP < 80 mmHg) and received one or more HEMS interventions (Table 4). Fourteen patients presenting to the HEMS team with hypovolemic shock were not anesthetized and were cerebrating well, and therefore, as per AAKSS SOP, did not receive blood products from the attending HEMS team. All patients presenting with significant respiratory distress due to a pneumothorax received a pre-hospital chest drain. The majority of patients without circulatory- or ventilatory compromise did not need HEMS interventions. Nine patients, however, initially presented with stable physiology but needed HEMS interventions on route to hospital. On case review, 4 patients required blood product transfusion, either PRBC and/or FDP, 3 required placement of an intercostal chest drain, 1 patient required PHEA and 1 patient deteriorated to TCA requiring a RT and blood product transfusion.

**Table 4:** Presenting physiology of patients requiring HEMS-specific interventions for penetrating torso injuries

Presenting physiology	Total number of patients	Patients received interventions	Total number of interventions
	n[%]	n[%]	n[%]
Total	363	54	121
Uncompromised circulation and ventilation	272 [74.9]	9 [16.7]	13 [10.7]
Compromised circulation			
*TCA*	34 [9.4]	27 [50.0]	70 [57.9]
*SBP < 80 mmHg*	30 [8.3]	16 [29.6]	36 [29.8]
Compromised ventilation (without compromised circulation)			
*Isolated RR > 30 or SpO*_*2*_ *< 92%*	27 [7.4]	2 [3.7]	2 [1.6]

*TCA* traumatic cardiac arrest; *SBP* systolic blood pressure; *RR* respiratory rate

In univariate correlation analysis, male gender (*r* = .248, *p* < .001), TCA (*r* = .513 *p* < .001), presence of hypotension- (*r* = .303, *p* < .001), tachycardia (*r* = .286 (*p* < .001), reduced oxygen saturation (< 92%) (*r* = .288, *p* = .001) and respiration rate (*r* = .408, *p* = .001) were associated with one or more HEMS interventions being performed.

In multivariable logistic regression analysis TCA (OR 1.751 [1.41–2.16], hypotension (OR 1.40 [1.18–1.67] and SpO_2_ < 92% (OR 1.39 [1.17–1.65] were independent physiological findings associated with the requirement of HEMS interventions on scene or on route to hospital.

## Discussion

Penetrating trauma has been a substantial part of the trauma workload in urban areas in the UK for the last decade [20]. However, the incidence of penetrating trauma is increasing in both rural and semi-rural regions. In this observational study performed in a semi-rural HEMS service, we demonstrate that around 15% of the patients with penetrating torso injuries attended by HEMS require one or more HEMS specific time-critical interventions in the pre-hospital setting, whereas 85% do not require interventions. Unsurprisingly, presenting physiology was associated with the need for HEMS interventions. The majority of the patients in traumatic cardiac arrest received HEMS interventions, whereas HEMS interventions were performed in around 50% of the patients presenting with hypovolemic shock physiology, and in all patients presenting with isolated severe respiratory distress without hypotension.

In this study, we report a unique geographical distribution of penetrating torso trauma cases attended by HEMS, largely aligning to the drug-trafficking county lines [21, 22]. The multi-agency Kent and Medway Gang Strategy (2018–2021) warn of steadily increasing gang operations across the Kent and Medway region, originating from London-based individuals venturing into the region for ‘homegrown’ gangs. Given the proximity to London, the counties of Surrey and Sussex (predominantly in those areas with the highest socioeconomic deprivation) are liable to the same trend [23, 24]. An appreciation of the geographical distribution of penetrating trauma in relation to diurnal patterns is imperative to not only multi-agency knife violence prevention and education but also policing and safeguarding the needs of the vulnerable [25, 26]. AAKSS considers this evidence-base essential from which to contribute valuable preventative educational strategies.

Interestingly, we could not establish an association between the time elapsed from 999/112 call to attendance and the need for HEMS interventions. This likely reflects the spectrum of pathology resulting from penetrating injuries. Most patients do not have significant injuries to vascular structures or vital organs and will remain cardiovascularly stable until presentation at hospital. A small group will have major arterial injuries and will almost universally need HEMS interventions on scene, even when they are seen very shortly after their injury. Finally, a third group will have ongoing venous bleeding, with a potential to deteriorate in the pre-hospital phase of their injury. It is likely that these patients are underrepresented in the current cohort, and thereby obscure any association between the time elapsed from 999/112 call to HEMS attendance and the need for HEMS interventions.

In our study population, 9 patients initially presented without significant haemodynamic or ventilatory compromise, but deteriorated during transit to hospital and required advanced medical intervention, ranging from inter-costal chest drain insertion or blood product transfusion to PHEA and resuscitative thoracotomy. Although this is a small percentage of all patients presenting without significant haemodynamic or ventilatory compromise, the interventions performed may be lifesaving in nature given the long average distance from the nearest MTC.

A certain degree of over-triage of patients initially presenting without significant hemodynamic- or ventilatory compromise by HEMS teams in this respect, is almost inevitable. Previous studies have demonstrated that the accuracy of clinical examination to establish the degree of bleeding and potential for internal damage after penetrating injury is poor [14, 27, 28]. Our clinical judgement in these patients is often obscured, as the scene of the incident is often not witnessed (making it hard to estimate the amount of blood loss), and the physiological response to blood loss in the patients may differ from non-penetrating injuries. Especially with peritoneal breeching, relative bradycardia may be present, obscuring the normal baroreceptor response. Therefore, all torso stab wounds should be considered as invasive until imaging is complete [14] and HEMS teams should have a low threshold to escort these patients to hospital, irrespective of their physiological state. However, given the small proportion of hemodynamically stable patients who need a HEMS intervention, the clinical team must perform a risk assessment, to include potential for clinical deterioration. The outcome of such a risk assessment is likely dependant on: availability of HEMS and/or other critical care teams, suspected transport time to hospital, and exact anatomical location of injury. There is also a trend towards avoiding pre-hospital anaesthesia in this group of patients, in favour of blood product transfusion and rapid transport to hospital. Early intubation in the hypovolaemic patient, particularly the switch to positive pressure ventilation, can lead to rapid cardiovascular collapse in some patients.

Over half of the penetrating trauma incidents attended were within a night operational context. This is in line with previous work reporting an increased rate of victims of assault and self-harm compared to daylight hours [29]. Night-time availability of HEMS and the availability of ad-hoc or pre-surveyed sites contributes to expedited care for these patients [29]. In the present study however, the latter could not be demonstrated as during the study period helipad access to the MTC’s was restricted to daylight operation hours only, and therefore patients were universally ground escorted by the HEMS teams during night operational hours.

HEMS teams are likely not only provide advanced clinical skills, but also to support advanced clinical decision-making in terms of triage and transport. This is hard to substantiate and not measured in the current study. However, Cowley et al. reported that patients with penetrating injuries presenting in the region of our HEMS service were more likely triaged to an MTC when attended by an enhanced care team compared to standard ground ambulance crews (OR 7.59, 3.70–15.37, *p* < 0.0001) [4] . Although this could be attributed to a selection bias regarding injury burden and injury severity, it is likely that due to clinical experience these teams are perhaps increasingly aware of underlying injuries, and the potential for deterioration on route to hospital. Enhanced pre-hospital care practitioners may expedite transport to hospital, even when ground escorting patients, as they have the capability to intervene during transit if necessary.

### Limitations

Our study has several limitations, mostly inherent to the study design. First, our population studied is confined to patients attended by HEMS, and not to the wider population of patients with penetrating torso injuries. However, as penetrating torso injury is a dispatch criterium for AAKSS HEMS, it is unlikely that many patients have been missed during the study period. Further, our study results cannot automatically be extrapolated to other geographical regions with different transport times and/or availability of 24/7 HEMS. We are conscious that nearly half of the HEMS interventions are performed in those patients pronounced life extinct. Finally, we have no information on the definitive injuries and/or outcome of our patients. Therefore, it remains speculative that the HEMS interventions performed have contributed to a favourable outcome in our patients, or if those transported in a hemodynamically stable condition without HEMS interventions possibly deteriorated at a later stage. The dispatch elements, data from the ground ambulance prior to arrival of HEMS and long term patient outcome were beyond the reach of this initial study, however this is planned for a future project.

## Conclusion

HEMS teams provide a potentially important contribution to the pre-hospital treatment of patients with penetrating torso injuries in rural and semi-rural areas, especially when they present with unstable physiology. A certain degree of over-triage is inevitable in these patients, as it is hard to predict which patients will deteriorate on route to hospital and will need HEMS interventions. The results of this study showing a potentially predictable geographical dispersion of penetrating trauma could inform multi-agency knife crime prevention strategy.

## Data Availability

The datasets used and/or analysed during the current study are available from the corresponding author on reasonable request.

## Abbreviations

HEMS: Helicopter emergency medical service
UK: United Kingdom
AAKSS: Air ambulance kent surrey sussex
MTC: Major trauma centre
GCS: Glasgow coma scale
SBP: Systolic blood pressure
TCA: Traumatic cardiac arrest
SpO_2_: Oxygen saturation
EMS: Emergency medical services
FiO_2_: Fraction of inspired oxygen
PRBC: Packed red blood cells
FDP: Freeze-dried plasma
TU: Trauma unit
PHEA: Pre-hospital emergency anaesthesia
RT: Resuscitative thoracotomy
GE: Ground escort
GA: Ground assist
PLE: Pronounced life extinct
HR: Heart rate
RR: Respiratory rate
LEH: Local emergency hospital
